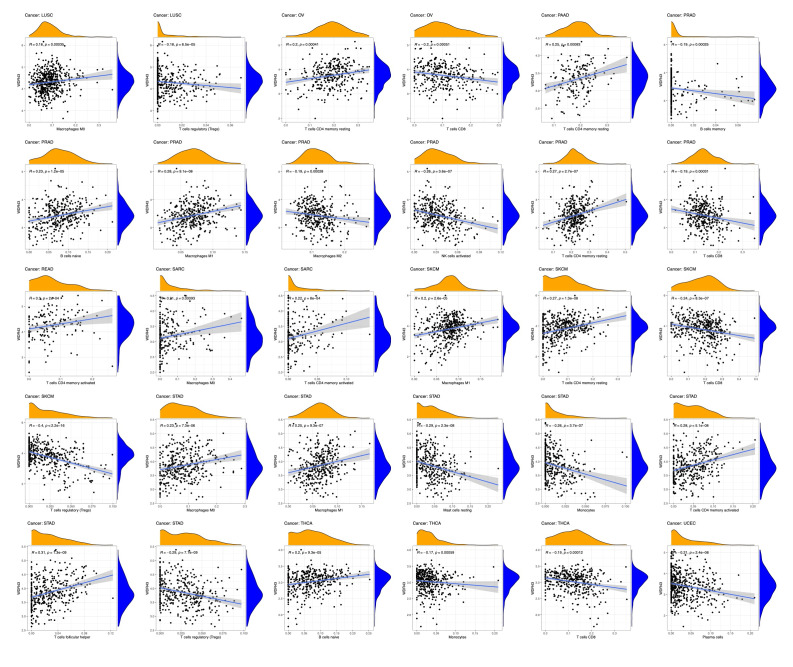# WD repeat domain 43 as a new predictive indicator and its connection with tumor immune cell infiltration in pan-cancer

**DOI:** 10.1097/MD.0000000000039153

**Published:** 2024-08-02

**Authors:** Xin Yang, Ting Luo, Zhixin Liu, Jiao Liu, Zhuo Yang

**Affiliations:** a Department of Digestive Endoscopy, General Hospital of Northern Theater Command, Shenyang, China.

**Keywords:** hepatocellular carcinoma, immune infiltration, pan-cancer, prognosis, WDR43

## Abstract

**Background:**

WD repeat domain 43 (WDR43) is a protein component that encodes WD-repeats and is involved in ribosome biogenesis. However, little is known about the role of WDR43 in cancer prognosis and immune modulation.

**Methods:**

In this study, we analyzed the expression and prognostic significance of WDR43 in pan-cancer using the Cancer Genome Atlas, the Genotype-Tissue Expression, and the Human Protein Atlas. We also examined the differential expression of WDR43 in liver hepatocellular carcinoma (LIHC) and adjacent tissues of 48 patients using immunohistochemistry. Additionally, we investigated the correlation between WDR43 and clinical characteristics, gene alterations, tumor mutation burden, microsatellite instability, mismatch repair, tumor microenvironment, immune infiltrating cells, and immune-related genes using bioinformatics methods. Gene set enrichment analysis was conducted, and potential biological mechanisms were identified.

**Results:**

Immunohistochemistry staining showed that WDR43 was overexpressed in LIHC among 48 patients. Upregulation of WDR43 was associated with unfavorable prognosis, including overall survival in various types of cancer such as LIHC, uterine corpus endometrial cancer, head and neck squamous cell carcinoma, and pancreatic adenocarcinoma. Differential expression of WDR43 was significantly correlated with microsatellite instability, mismatch repair, and immune cell infiltration. Gene ontology annotation analysis revealed that these genes were significantly enriched in immune-related functions, including immune response, immune regulation, and signaling pathways.

**Conclusion:**

We conducted a thorough investigation of the clinical features, phases of tumor development, immune infiltration, gene mutation, and functional enrichment analysis of WDR43 in various types of cancer. This research offers valuable insight into the significance and function of WDR43 in clinical therapy.

## 1. Introduction

Current evidence suggests that cancer incidence and mortality rates are increasing every year, posing a significant threat to global public health.^[1,2]^ Despite significant progress in cancer detection and therapy, most malignancies have a poor 5-year overall survival (OS) rate.^[2]^ Cancers also impose a significant financial burden on society worldwide.^[3]^ Therefore, there is a critical need for novel cancer diagnostic and therapeutic approaches. The liver ranks as the sixth most frequent location for primary tumors in humans and is the fourth highest contributor to cancer-related mortality worldwide.^[2]^ Most liver malignancies, about 90%, are caused by hepatocellular carcinoma.^[4]^ Although immunotherapy and gene-targeted therapy are promising therapeutic modalities, their efficacy remains poor.^[5]^ Furthermore, despite the development of new treatments and advances in medicine, the survival rate and prognosis of cancer patients continue to be unsatisfactory. This can be attributed to drug resistance, as well as the complex nature of cancer onset, progression, and prediction.^[2]^

The complex and multistep nature of tumorigenesis has been well-established. The tumor microenvironment (TME), which comprises a large degree of immune cell infiltration, has been demonstrated to have a vital role in the activation of oncogenes and the production of aberrant proteins and stress signals in human malignancies.^[6,7]^ Therefore, it is necessary to research novel sensitive tumor biomarkers and their ability to interact with cancer to develop an effective cancer immunotherapy.

Each member of the WD repeat protein family possesses a cis-repeat WD motif that ranges from 4 to 16 repeats. In the P subunit of G proteins, the WD motif protein, now known as WD40, was discovered for the first time.^[8,9]^ Over the years, WDR domains have been found in approximately 610 confirmed or predicted human proteins.^[10]^ As a significant member of the WDR protein group, the WD repeat domain 43 (WDR43) gene is associated with ribosome biosynthesis and craniofacial development defects caused by ribosomal disorders.^[11]^ A recent study discovered that the c-MYC-WDR43 signaling pathway enhances chemoresistance and tumor growth in colorectal cancer (CRC) by reducing p53 activation.^[12,13]^ Additionally, another study found that direct interaction between WDR43 and cyclin-dependent kinase 2 promotes cyclin expression, suggesting that WDR43 may be a potential therapeutic target for non-small cell lung cancer (NSCLC).^[14]^ WDR43 gene has been linked to poor outcomes in cervical cancer, breast cancer, and CRC.^[15]^ All of these studies suggest that WDR43 is associated with cancer progression. However, the expression of WDR43 in liver hepatocellular carcinoma (LIHC) and its precise function in pan-cancer are still unknown.

This study utilized bioinformatics tools to investigate the relationship between WDR43 expression levels and cancer prognosis and the connection between WDR43 expression and tumor immunity by analyzing WDR43 expression levels in multiple cancer types. The results revealed that WDR43 is a promising biomarker correlated with tumor detection and response to immunotherapeutic agents.

## 2. Materials and methods

### 2.1. Immunohistochemical staining and scoring

A LIHC tissue microarray (D097Lv01) consisting of 48 tumor tissues and 48 corresponding normal tissues was purchased from Xi’an Bioaitech Co., Ltd (http://www.bioaitech.com). This microarray was used to measure the protein expression level of WDR43 through immunohistochemistry (IHC). The anti-WDR43 antibody (abs10340; Absin, Shanghai, China) was used in the experiment, which was conducted following the manufacturer’s instructions. Immunohisto-cytochemistry was conducted as previously described.^[16]^ Details information on the criteria for grading the WDR43 protein level was as follows: following is a list of the criteria that were used to score the levels of WDR43 protein: the degree of staining was rated as 0 (no staining), 1 (light yellow), 2 (dark yellow), or 3 (brown); the percentage of positively stained cells was scored as 0 (<1%), 1 (1%–25%), 2 (26%–50%), 3 (51%–75%), or 4 (76%–100%). The ultimate IHC score, which ranges from 0 to 12, was determined by multiplying the score for staining intensity with the score for percentage.^[17]^

### 2.2. Differential expression analysis and data processing

The Tumor Immune Estimation Resource (TIMER) database (https://cistrome.shinyapps.io/timer/) is a web facility that contains a collection of 10,104 samples derived from 33 distinct cancer forms sourced through the dataset provided by The Cancer Genome Atlas (TCGA). This resource evaluated the differential levels of WDR43 expression in malignant tissues and healthy tissues across various cancers. Immunohistochemistry pictures of normal and malignant human tissues were obtained from the Human Protein Atlas (HPA) dataset (https://www.proteinatlas.org/). Additionally, the Gene Expression Profiling Interactive Analysis database was used to examine the clinical staging of WDR43 in various tumor tissues obtained from the Genotype-Tissue Expression (GTEx) and TCGA datasets (GEPIA2, http://gepia2.cancer-pku.cn/#index). mRNA expression of WDR43 in malignant tissues and healthy tissues was compared using specimens from TCGA.

Moreover, paired samples from TCGA were used to compare different expression levels of WDR43 in healthy tissue and tumor tissue within the same individuals. Furthermore, results from the Clinical Proteomic Tumor Analysis Consortium (CPTAC) and HPA databases were used to investigate differential expression levels of WDR43 protein across various cancers. Ethical approval and patient consent were exempted as this investigation followed the established protocols of TCGA and GTEx datasets.

### 2.3. Survival analysis

TCGA has been utilized to extract survival and clinical data for various tumors. Then, our study examined the association among WD43 expression levels and the individual’s prognosis across 33 distinct cancer types, utilizing forest plots within Kaplan–Meier (KM) curves in KM Plotter (https://kmplot.com/analysis/), UCSC Xena Shiny (https://github.com/openbiox/UCSCXenaShiny), and GEPIA2. Survival studies were conducted employing KM and forest plot curves.

### 2.4. Establishment and evaluation of the nomogram models

Herein, we constructed a nomogram model to independently predict cancer prognosis based on clinical characteristics and patients’ risk scores. We evaluated the consistency between predicted and observed outcomes using calibration curve analysis. The consistency index (C index) assessed the accuracy of the nomogram’s predictions. The receiver operating characteristic (ROC) curve of time-dependent curve of diagnosis, nomographs, and calibration charts was generated using the R (version 6.2-0) package RMS and time ROC.

### 2.5. Genetic mutation analysis

This study used the open-access web facility cBioPortal (https://www.cbioportal.org/) to investigate, visualize, and examine multidimensional cancer genomics data.

Subsequently, we obtained information on the genetic alterations of WDR43, including mutational types, copy number alterations, detailed alteration frequencies in every TCGA cancer type, and information on the mutation site from the website. We then evaluated the statistical significance of survival variations among all TCGA tumors with or without WDR43 genetic modifications by computing log-rank *P* values and KM curves.

### 2.6. Relationship between expression of WD repeat domain 43 and microsatellite instability, tumor mutational burden, and mismatch repair gene expression

The current investigation obtained and analyzed genetic mutation information for 33 distinct forms of cancer from the TCGA database. We determined the tumor mutational burden (TMB) of every specimen using Perl. We generated a radar map using the “ggradar” R package to examine the connection between the differential expression of WDR43 protein and TMB and microsatellite instability (MSI) using the Spearman correlation test.

Using expression profile data from TCGA, we evaluated the different levels of expression of the MutS homolog 6 (MSH6), MutS homolog 2 (MSH2), MutL homologous gene (MLH1), postmeiotic segregation increased 2 (PMS2), and epithelial cell adhesion molecule (EPCAM) in various cancers. We also determined the association between mismatch repair (MMR) levels and WDR43 gene expression. We generated heatmaps of the data using the R package “ggplot.”

### 2.7. Tumor microenvironment and checkpoint gene analysis

Herein, we calculated immunological and stromal scores using the “ESTIMATE” R package. Spearman correlation analysis was also performed to investigate the connection between WDR43 and the immune and stromal scores. Then, we examined WDR43 expression within the TCGA database and its association with systemic immune checkpoint blockade gene expression using the “Gene_Corr” module of the TIMER2.0 database. The generated heatmap is statistically significant. After obtaining Spearman’s rho values and corresponding *P* values with purity adjustment, we used the R language’s “ggplot2” package (version 3.3.3) to generate the relevant heatmaps.

### 2.8. Immune infiltration analysis

In this study, we utilized the ssGSEA algorithm from the R package “GSVA” (version 1.34.0) to explore the association between WDR43 and 24 distinct categories of immune infiltrating cells in various forms of cancer. The R package “ggplot2” (version 3.3.3) was used to visualize the data. The TIMER database demonstrated a connection between WDR43 gene expression and pan-cancer immune invasion abundance. Various algorithms, including CIBERSORT, CIBERSORT ABS, EPIC, MCPCOUNTER, TIMER, QUANTISEQ, and XCELL, were used to evaluate immunological infiltration of immune cells within TIMER2.0 in all tumors, including CD4 + T cells, CD8 + T cells, macrophages, NK cells, neutrophils, endothelial cells, bone marrow-derived suppressor cells, and cancer-related fibroblasts. The *P* value and cross-sectional association value were computed using a purity-adjusted Spearman rank correlation test. Heatmaps were used to represent the data for all immune cells. Scatter plots also represented the relationship between cancer-related fibroblasts and macrophages and the expression of WDR43.

WDR43 co-expression and immune-associated genes, such as genes encoding major chemokines, chemokine receptors, immune activation, immunosuppression, and histocompatibility complex (MHC) proteins, were examined and visualized using the R package “ggplot2” (version 3.6.3).

### 2.9. WDR43-associated gene enrichment analysis

The protein–protein interaction networks of the top 50 WDR43-binding proteins were acquired using the STRING website. The top 100 associated genes were compiled using the cBioPortal database. Pearson correlation analysis was performed on the 6 most highly correlated genes with WDR43. A scatter plot was generated to depict the Log2 (TPM), correlation coefficient *R* value, and *P* value. Next, the “Gene_Corr” section in the Timer2.0 database was used to input the 6 genes with the greatest association and generate a correlation heatmap. Venn plots of both database genes were created using the ggplot2 package in R. Enrichment analyses based on the Kyoto Encyclopedia of Genes and Genomes (KEGG) and gene ontology (GO) were conducted using the clusterProfiler package (version 3.14.3). A scatter plot was generated to depict the Log2 (TPM), correlation coefficient *R* value, and *P* value, with a *P* value < .05 deemed statistically significant.

### 2.10. Gene set enrichment analysis

After separating the TCGA data into groups with high and low differential expression of WDR43, differentially expressed genes associated with WDR43 were obtained. The R packages “limma (version 3.44.3),” “org.Hs.eg.db (version 3.11.4),” “clusterProfiler (version 3.16.1),” and “enrichplot (version 1.8.1)” were used to perform functional analysis.

### 2.11. Statistical analysis

The data normalization for the gene expression process was conducted using log2 transformation. Two sets of *t* tests were performed to compare healthy and malignant tissue. A *P* value < .05 was considered statistically significant.

Herein, we utilized the Cox proportional hazard regression model, log-rank test, and KM curve for survival analyses. The connection between the 2 variables was investigated using either Spearman or Pearson correlation, with a statistical significance set at *P* value < .05.

All statistical analyses were performed using R software (version 4.0).

## 3. Results

### 3.1. WDR43 expression analysis

**Figure 1.**
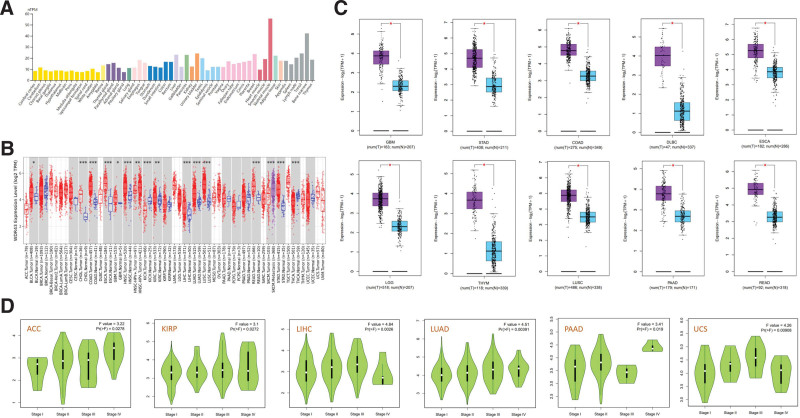
The expression of WDR43 in normal tissues, cancers, and pathological stages. (A) WDR43 differential expression in various healthy tissues obtained from the HPA database. (B) The differential expression of WDR43 in various malignancies (red) and healthy tissues (blue) from the TCGA database by TIMER. (C) The expression variance of WDR43 among the tumor (purple) and healthy tissues (blue) in COAD, GBM, STAD, DLBC, ESCA, LGG, THYM, LUSC, PAAD, READ based on GTEx and TCGA database utilizing GEPIA2. (D) Association among WDR43 differential expression and the main pathological phases of ACC, KIRP, LIHC, LUAD, PAAD, and UCS (GEPIA2). *P < .05; **P < .01; ***P < .001. ACC = adrenocortical carcinoma, COAD = colon adenocarcinoma, DLBC = diffuse large B-cell lymphoma, ESCA = esophageal carcinoma, GBM = glioblastoma multiforme, GEPIA2 = Gene Expression Profiling Interactive Analysis version 2, HPA = Human Protein Atlas, KIRP = kidney renal papillary cell carcinoma, LGG = lower grade glioma, LIHC = liver hepatocellular carcinoma, LUAD = lung adenocarcinoma, LUSC = lung squamous cell carcinoma, PAAD = pancreatic adenocarcinoma, READ = rectum adenocarcinoma, STAD = stomach adenocarcinoma, TCGA = The Cancer Genome Atlas, THYM = thymoma, TIMER = tumor immune estimation resource version, UCS = uterine carcinosarcoma, WDR43 = WD repeat domain 43.

Initially, our objective was to identify the levels of expression of WDR43 in various healthy tissues from healthy individuals using the HPA database, based on a consensus dataset created by integrating data obtained from 3 transcriptome datasets (HPA, GTEx, and function annotation of the mammalian genome 5). The level of WDR43 expression varied in various tissue types, with significantly high expression in skeletal muscle and bone marrow, as demonstrated in Figure 1A. WDR43 expression was extracted from 33 samples containing malignant and healthy tissues within the TCGA database using the Timer2.0 website. According to Figure 1B, the upregulation of WDR43 expression has been detected through diverse forms of cancer, involving cholangiocarcinoma, esophageal carcinoma (ESCA), lung squamous cell carcinoma (LUSC), colon adenocarcinoma (COAD), lung adenocarcinoma (LUAD), head and neck squamous cell carcinoma (HNSC), uterine corpus endometrial carcinoma (UCEC), LIHC, stomach adenocarcinoma (STAD), and rectum adenocarcinoma (READ) (all *P < *.001), kidney renal clear cell carcinoma (KIRC) (*P < *.01), bladder urothelial carcinoma (BLCA) and glioblastoma (GBM) (*P < *.05). Intriguingly, WDR43 differential expression has been discovered to exhibit significant reduction within tissues of thyroid carcinoma (THCA) and kidney chromophobe (KICH) as compared to control tissues. Figure 1B illustrates specific types of cancer that either lack or have limited normal tissue samples. Using GEPIA2, normal tissues were utilized as controls from the GTEx dataset for certain cancer types (Fig. 1C). Our findings revealed that compared with the normal controls, WDR43 expression exhibited a significant increase within tumors, including COAD, GBM, STAD, ESCA, pancreatic adenocarcinoma (PAAD), lower grade glioma (LGG), diffuse large B-cell lymphoma (DLBC), thymoma, LUSC, and READ (Fig. 1C, *P* < .05).

**Figure 2.**
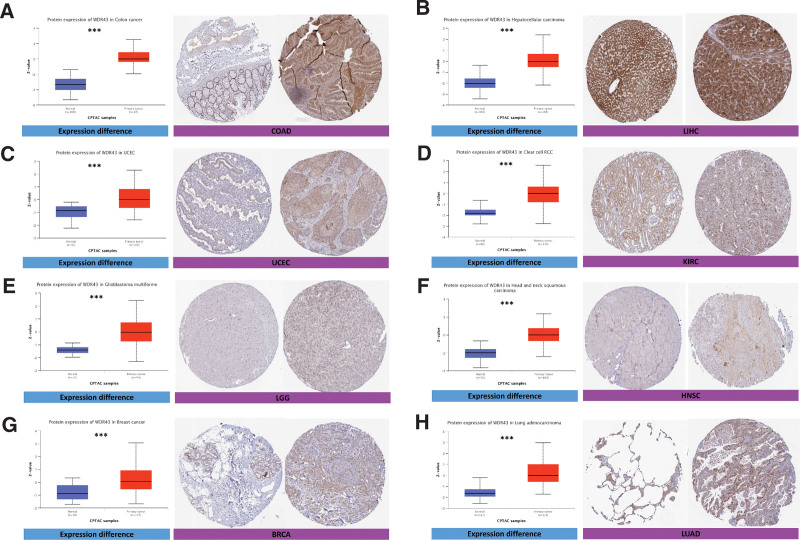
WDR43 protein expression in tumor and normal tissues. WDR43 protein expression in tumor and normal tissues on CPTAC and HPA platforms, respectively, including (A) COAD, (B) LIHC, (C) UCEC, (D) KIRC, (E) LGG, (F) HNSC, (G) BRCA, and (H) LUAD. BRCA = breast invasive carcinoma, COAD = colon adenocarcinoma, CPTAC = Clinical Proteomic Tumor Analysis Consortium, HNSC = head and neck squamous cell carcinoma, HPA = Human Protein Atlas, KIRC = kidney renal clear cell carcinoma, LGG = lower grade glioma, LIHC = liver hepatocellular carcinoma, LUAD = lung adenocarcinoma, UCEC = uterine corpus endometrial carcinoma, WDR43 = WD repeat domain 43.

The protein expression of WDR43 exhibited a statistically significant increase in UCEC, COAD, LUAD, LGG, HNSC, breast invasive carcinoma, renal papillary cell carcinoma (KIRP), and tumor tissue compared to para-cancerous tissue (Fig. 2), based on online data obtained from CPTAC datasets.

**Figure 3.**
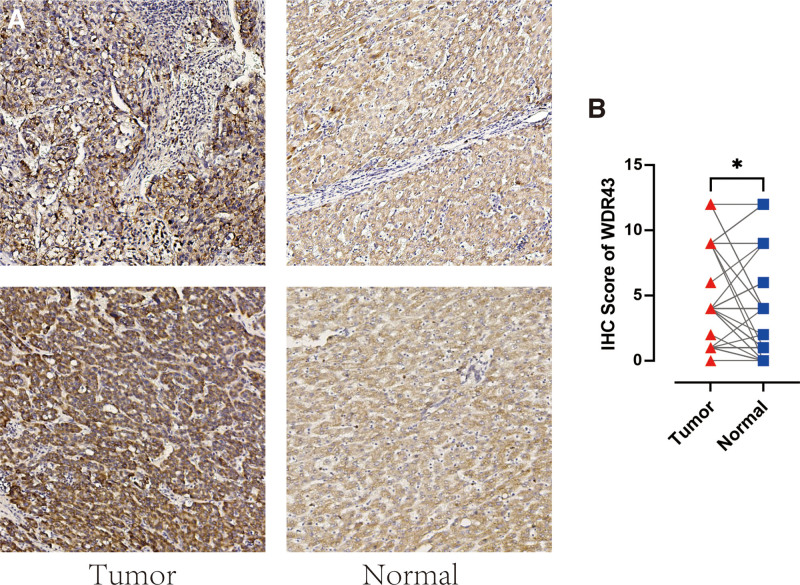
Expression of WDR43 in LIHC by IHC. (A) The protein expression of WDR43 in the matched tumor and normal tissues of LIHC was detected by immunohistochemistry staining. (B) Quantitation of MDR43 expression in the LIHC tumor tissues and normal tissues shown as IHC Score (n = 48). *P < .05. IHC = immunohistochemistry, LIHC = liver hepatocellular carcinoma, WDR43 = WD repeat domain 43.

Additionally, IHC labeling was conducted on 48 pairs of malignant and normal tissues in LIHC to validate the protein-level expression of WDR43 in LIHC. Figure 3 demonstrates a notable increase in the expression of WDR43 in LIHC tumor tissue contrasted to the corresponding normal tissue (Fig. 3A) (*P* < .05, Fig. 3B). The results of this research align with the data obtained from the TCGA and CPTAC datasets available online.

Furthermore, the utilization of GEPIA2 datasets revealed a significant connection between WDR43 expression levels and various pathological stages across multiple cancer forms, such as adrenocortical carcinoma (ACC), LIHC, LUAD, KIRP, PAAD, and uterine carcinosarcoma (Fig. 1D).

### 3.2. The potential connection between the differential expression of WDR43 and prognosis within pan-cancer

For investigating the connection among clinical outcomes and WDR43 expression in pan-cancer, a KM survival analysis has been conducted to examine the predictive significance of WDR43, such as progression-free interval (PFI), disease-specific survival (DSS), OS, and recurrence-free survival (RFS).

**Figure 4.**
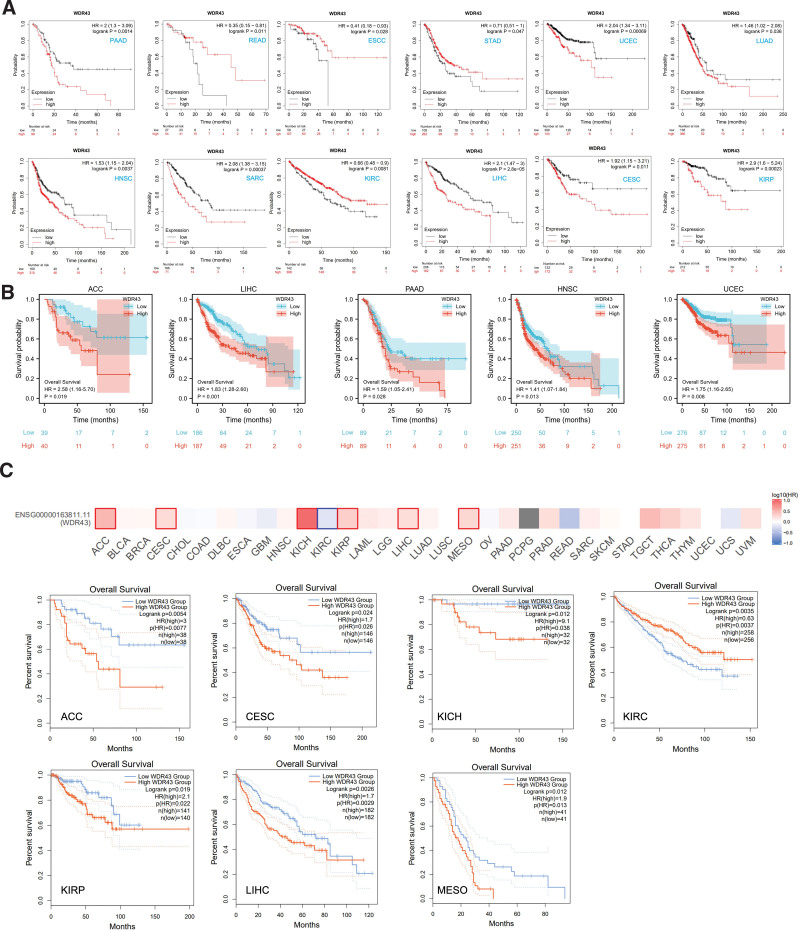
Association between the expressions of WDR43 in individuals with OS. (A) Verification of the OS curve of WDR43 in KM. (B) Validation of the OS curve of WDR43 from the TCGA database. (C) The heatmap and KM curve of OS obtained from the GEPIA2 database. Values of *P* < .05 were documented and presented. GEPIA2 = Gene Expression Profiling Interactive Analysis version 2, KM = Kaplan–Meier, OS = overall survival, TCGA = The Cancer Genome Atlas, WDR43 = WD repeat domain 43.

First, we utilized 3 datasets to investigate the association between the differential expression of WDR43 and OS within 33 distinct tumor types. The KM analysis outcomes exhibited that the overexpression of WDR43 was correlated with shorter OS in PAAD, UCEC, LUAD, HNSC, sarcoma (SARC), LIHC, KIRP, and cervical and endocervical cancer (CESC). In contrast, the upregulation of WDR43 in READ, STAD, esophageal squamous cell carcinoma, and KIRC correlated with longer OS (Fig. 4A). Additionally, based on TCGA data, Cox proportional hazards model analysis was conducted to assess the predictive significance of the WDR43 in various forms of cancer. According to Figure S1A, Supplemental Digital Content, http://links.lww.com/MD/N284, and Figure 4B, the expression of WDR43 exhibited a negative connection with OS in ACC, DLBC, KICH, HNSC, LIHC, and PAAD patients. Furthermore, in GEPIA2, overexpression of WDR43 was correlated with unfavorable OS in ACC, CESC, KICH, KIRP, LIHC, and mesothelioma (MESO) but correlated with positive OS in KIRC (Fig. 4C).

Additionally, we evaluated the DSS data (Figure S1B, Supplemental Digital Content, http://links.lww.com/MD/N284) and found negative correlations between WDR43 expression and prediction in individuals diagnosed with ACC, KIRP, LIHC, MESO, and UCEC. However, the expression of WDR43 revealed a better association within KIRC. Concerning the associations between the expression of WDR43 and PFI (Figure S1C, Supplemental Digital Content, http://links.lww.com/MD/N284), we explored that high expression of WDR43 negatively affected patients’ PFI in pheochromocytoma and paraganglioma, UCEC, LIHC, KIRC, KIRP, ACC, mesothelioma and KICH. In contrast, a better association has been detected in PAAD. Eventually, RFS results obtained from Cox regression analysis indicated that WDR43 constituted a statistically significant risk factor for PAAD, LIHC, LUAD, LUSC, KIRP, and UCEC patients and a protective factor against BLCA, KIRC, ovarian serous cystadenocarcinoma (OV) and STAD patients (Figure S1D, Supplemental Digital Content, http://links.lww.com/MD/N284). These outcomes indicate that WDR43 expression affects the prediction of various forms of cancers, such as OS, DSS, PFI, and RFS.

### 3.3. Construction and assessment of nomogram models

**Figure 5.**
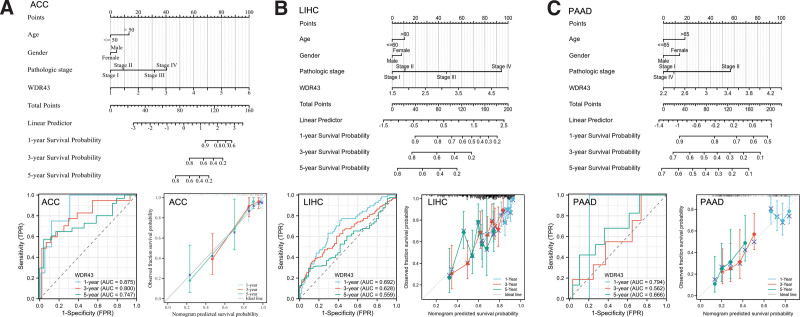
Nomogram models were developed and assessed. Development of a nomogram model incorporating WDR43 expression in ACC (A), LIHC (B), and PAAD (C). Time-dependent survival ROC curve analysis to predict 1-, 3- and 5-yr survival rates. The nomogram model was further assessed using 1-, 3-, and 5-yr calibration curves. ACC = adrenocortical carcinoma, LIHC = liver hepatocellular carcinoma, PAAD = pancreatic adenocarcinoma, ROC = receiver operating characteristic, WDR43 = WD repeat domain 43.

Furthermore, a univariate Cox regression analysis was conducted to explore the impact that the differential expression of WDR43 has on the prediction of pancancers in terms of OS. According to the findings of univariate Cox regression, ACC, HNSC, LIHC, and PAAD with a specimen size of more than 500 were chosen to establish a nomogram for confirming the prognostic value. Time-dependent survival ROC curve of WDR43 was created to predict 1-, 3-, and 5-year survival rates, which is considered suitable for prediction. Furthermore, the calibration curves were utilized to assess the precision of predictions at 1-, 3-, and 5-years. The findings demonstrated that WDR43 had a significant prognostic contribution and showed good prediction power for the OS of ACC (Fig. 5A), LIHC (Fig. 5B), and PAAD (Fig. 5C).

### 3.4. Genetic alteration analysis of WDR43

**Figure 6.**
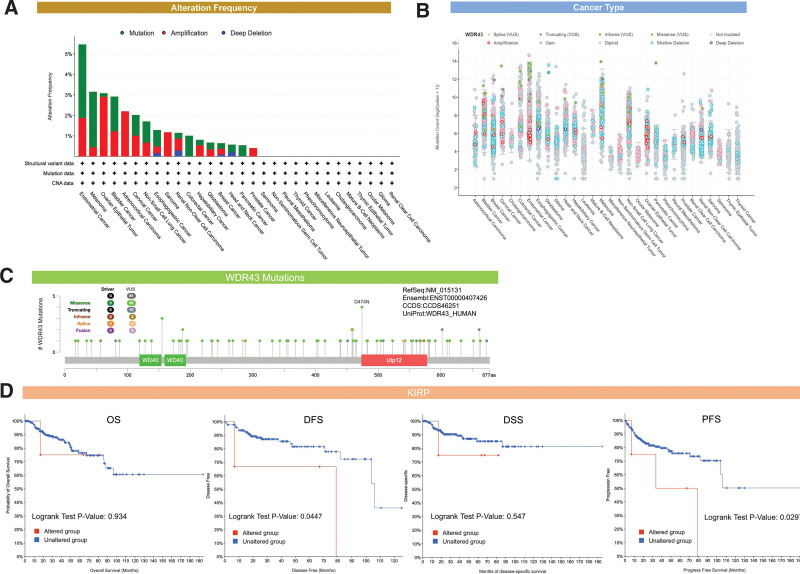
Gene alteration analysis of WDR43 in multiple cancers by cBioPortal. (A) A summary of the frequency of alterations observed in various forms of alterations across different types of cancer. (B) The types of WDR43 gene alterations and copy number in pan-cancer. (C) An overview of the various types, sites, and case quantity of MDR43 genetic modification. (D) The predictive significance of MMP7 genetic modification was investigated, and Kaplan–Meier curves of OS, DFS, DSS, and PFS in KIRP were generated. DFS = disease-specific survival, DSS = disease-specific survival, KIRP = kidney renal papillary cell carcinoma, MMP7 = matrix metalloproteinase-7, OS = overall survival, PFS = progression-free-survival, WDR43 = WD repeat domain 43.

cBioPortal was used to analyze genetic changes associated with WDR43. The findings showed that WDR43 exhibited the highest alteration frequencies in endometrial cancer (5.46%), with “mutation” (3.58%) and “amplification” (1.88%) being the 2 most common alteration types (Fig. 6A). Besides, “amplification” was the most common type in numerous cancers, including ovarian epithelial tumor, ACC, and SARC (>2%, >2%, and > 1% frequency, respectively). Figure 6B shows the detailed mutation type and copy number of WDR43 by cancer types, with data from 10,104 patients in TCGA, resulting in changes in gene expression. Figure 6C depicts the comprehensive forms, locations, and numbers for the genetic WDR43 alterations, and the most prevalent form of genetic modification in WDR43 is the missense mutation. Additionally, the WDR43 mutation, which was found in 3 instances of UCEC and 1 case of COAD, was related to most cases of D474N mutation, a missense mutation at Utp12 (Dip2/Utp12 Family domain containing 473–578aa) (Fig. 6C).

Furthermore, we determined whether there was an association between the genetic modification of WDR43 and prediction for all forms of TCGA cancer.

Unaltered WDR43 was recognized to have a worse outcome than the unaltered group in terms of disease-specific survival (*P = *.0447) and progression-free-survival (*P = *.0297), but not OS (*P = *.934) and DSS (*P = *.547) (Fig. 6D). No statistical significance variation was found within the prognosis observed among the groups of individuals with modified and unmodified WDR43 for the other TCGA cancers (data not shown).

**Figure 7.**
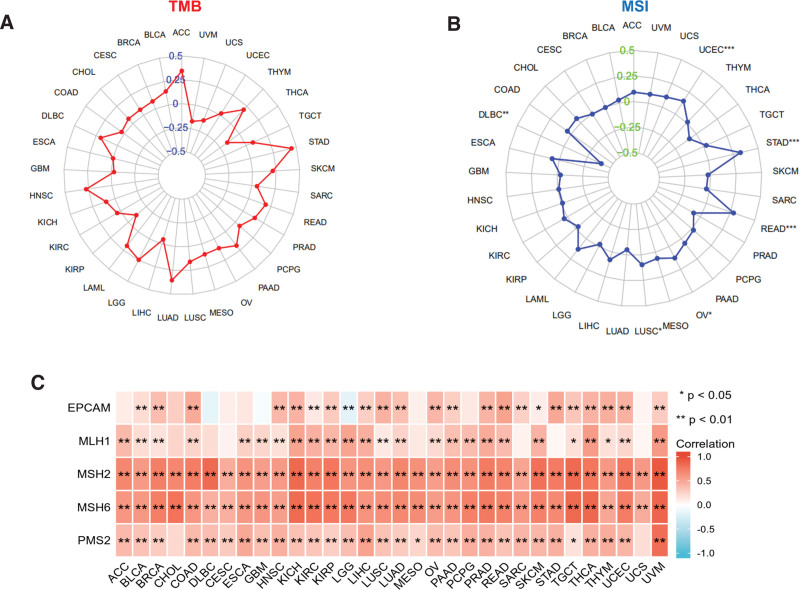
The connection between the expression of WDR43 and TMB, MSI, and MMR. (A) Radar plot representing the association between WDR43 expression and TMB in pan-cancer. The blue number demonstrates Spearman correlation coefficient. (B) Radar plot representing the correlation between WDR43 expression and MSI in pan-cancer. The green number shows Spearman correlation coefficient. (C) Heatmap showing the connection among WDR43 expression and 5 MMR genes (EPCAM, MLH1, MSH2, MSH6, and PMS2) in pan-cancer, in which red represents a positive correlation, and blue represents a negative correlation. EPCAM = epithelial cell adhesion molecule, MLH1 = MMR genes MutL homologous gene, MMR = mismatch repair, MSH2 = MutS homolog 2, MSH6 = MutS homolog 6, MSI = microsatellite instability, PMS2 = postmeiotic segregation increased 2, TMB = tumor mutation burden, WDR43 = WD repeat domain 43.

TMB denotes the number of DNA base mutations per million in tumor samples. MSI is distinguished by variations in microsatellite length, which occur because of the deletion or insertion of duplicate units in tumor versus normal tissues, leading to new microsatellite alleles. TMB and MSI are linked to cancer risk.^[18,19]^ We investigated the connection between WDR43 expression levels and TMB and MSI in multiple malignancies. According to Figure 7A, the association between WDR43 expression and TMB was not statistically significant among multiple tumors. Besides, overexpression of WDR43 exhibited a positive association with MSI in STAD, READ, OV, and LUSC; however, the expression of WDR43 exhibited negative association with MSI in DLBC (Fig. 7B).

The existence of a DNA repair mechanism known as MMR in cells is currently understood. When MMR genes are downregulated or functionally impaired, adequate repair of DNA replication errors is impossible, leading to a greater occurrence of somatic alterations.^[20]^ Our investigation performed an additional evaluation of the connection among the differential expression levels of WDR43 and 5 distinct MMR genes (MSH2, MSH6, PMS2, MLH1, and EPCAM) in 33 forms of cancer. As presented in Figure 7C, WDR43 expression demonstrated better association with the 5 different MMR genes in pan-cancer, except for gene EPCAM in LGG. The current investigation findings indicate that WDR43 may modulate tumor growth via modulating DNA mismatch repair.

### 3.5. TME and checkpoint gene analysis

**Figure 8.**
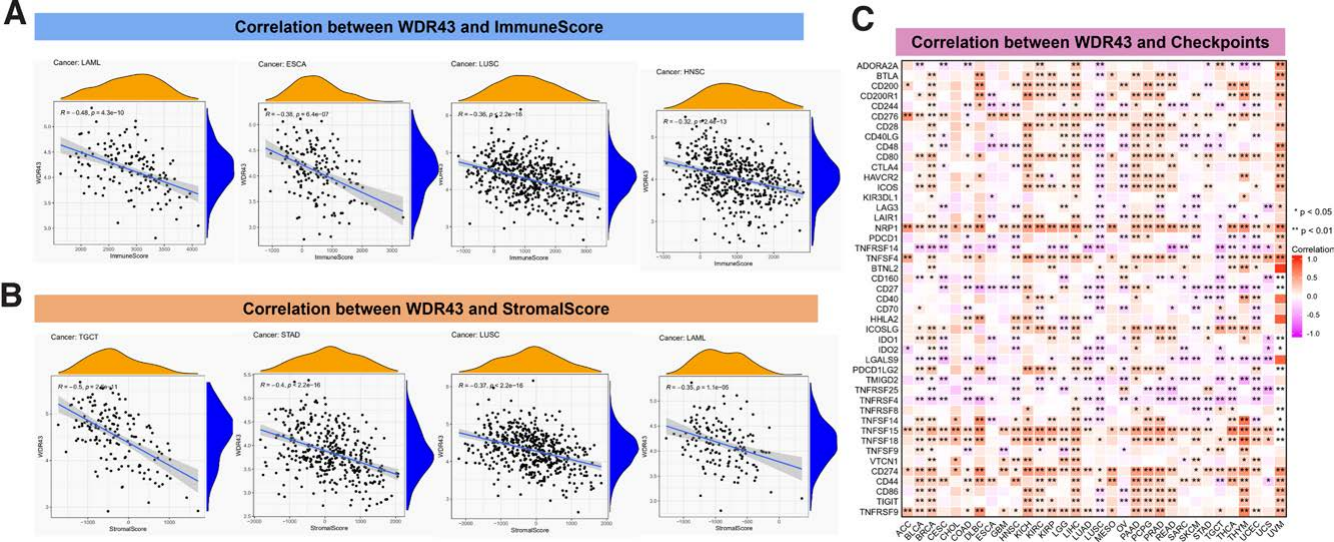
The expression of WDR43 is connected with the immune infiltration of tumors. Scatter plot of the connection among WDR43 expression with ImmuneScore (A) and StromalScore (B) in various cancers. (C) The connection between WDR43’s different expression levels and immune checkpoint-associated genes is that red represents a positive correlation, and blue represents a negative correlation. WDR43 = WD repeat domain 43.

The evidence suggests that TME significantly impacts the occurrence and advancement of cancers.^[21,22]^ Hence, exploring the connection between the different expression levels of WDR43 and TME is critical. We analyzed the association between the expression of WDR43 and the ImmuneScore and StromalScore within pan-cancer. In acute myeloid leukemia (*r* = −0.48, *P* = 4.3e-10), ESCA (*r* = −0.38, *P* = 6.4e-07), LUSC (*r* = −0.36, *P* < 2.2e-16), HNSC (*r* = −0.32, *P* = 2.4e-16), significant negative correlations among WDR43 expression and ImmuneScore were observed (Fig. 8A). Stromal scores in TGCT (*r* = −0.5, *P* = 2.6e-11), STAD (*r* = −0.4, *P* < 2.2e-16), LUSC (*r* = −0.34, *P* < 2.2e-16), acute myeloid leukemia (*r* = −0.35, *P* = 1.1e-05), skin cutaneous melanoma (*r* = −0.31, *P* = 7.2e-05) were negatively correlated with WDR43 expression (Fig. 8B).

We also determined the connection between WDR43 expression and immune checkpoint within 33 cancer forms (Fig. 8C). These outcomes demonstrated that WDR43 expression exhibited better association with CD274, CD276, NRP1, and TNFSF15 in various human malignancies. Besides, the expression of WDR43 exhibited a negative correlation with most immune checkpoint genes in LUSC.

### 3.6. Correlation among the levels of WDR43 expression and tumor immune cell infiltration

**Figure 9.**
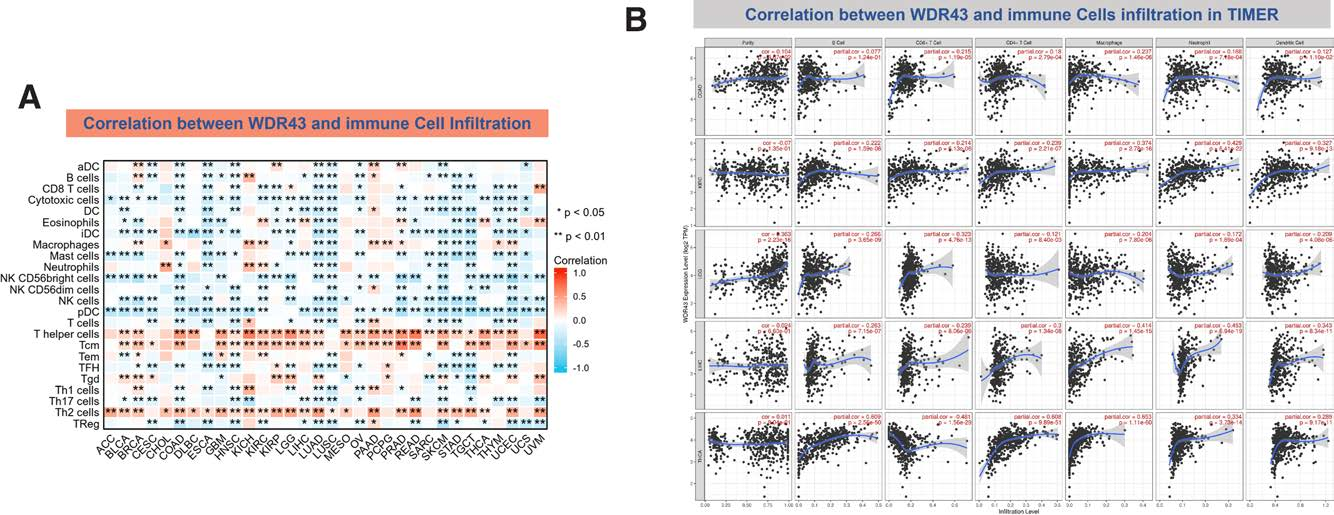
Correlation of WDR43 expression with Immune cell infiltration analysis. (A) Correlation between WDR43 expression and 24 immune-related cell infiltration in different cancers, in which red represents a positive correlation, and blue represents a negative correlation. (B) The connection between WDR43 expression and the infiltrations of immune cells in COAD, LGG, KIRC, LIHC, and THCA, respectively (TIMER database presented). COAD = colon adenocarcinoma, KIRC = kidney renal clear cell carcinoma, LGG = lower grade glioma, LIHC = liver hepatocellular carcinoma, THCA = thyroid carcinoma, TIMER = tumor immune estimation resource version, WDR43 = WD repeat domain 43.

There is an increasing body of evidence indicating that tumor-infiltrating immune cells may significantly affect patient survival outcomes.^[23]^ From this perspective, the connection between the differential expression of WDR43 within the occurrence of pan-cancer and the abundance of infiltration in 24 distinct subtypes of immune cells has been examined. In most malignancies, a significant association has been shown between the differential expression levels of WDR43 and immune cell infiltration level (Fig. 9A). An example is that the expression level of WDR43 demonstrated a favorable association with T helper cells, Tcm, and Th2 cells while displaying a poor association with NK cells, NK CD56bright cells, and pDC in pan cancers (Fig. 9A).

**Figure 10.**
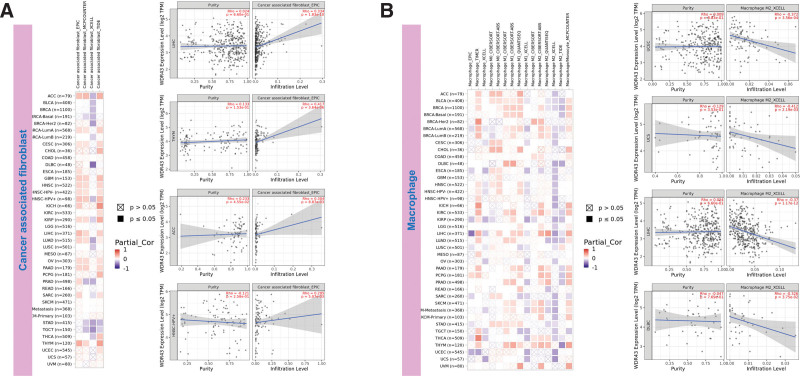
WDR43 expression was associated with infiltration of CAFs and TAMs. Based on EPIC, MCPCOUNTER, XCELL, and TIDE algorithms, heatmap and scatter map of the connection among WDR43 gene expression and CAFs infiltration level (A), TAMs infiltration level (B). CAFs = cancer-associated fibroblasts, TAMs = macrophages, WDR43 = WD repeat domain 43.

The top 5 tumors that exhibited the greatest association levels among the differential expression of WDR43 and the extent of the infiltration of immune cells included COAD, KIRC, LIHC, LGG, and THCA and were chosen for further investigation (Fig. 9B). Across the 5 tumors examined, the levels of WDR43 expression were favorably connected to several immune cells, for instance, CD8 + T cells, CD4 + T cells, B cells, macrophages, neutrophils and dendritic cells. The connection between the extent of WDR43 differential expression and the immune infiltration through distinct tumors in TCGA was then assessed utilizing the CIBERSORT, CIBERSORT abs, xCell, TIMER, MCP counter, quanTIseq, and EPIC algorithms. Our research findings were significantly associated with the immune infiltration levels and the differential expression of WDR43 within different malignancies (Figures S2, Supplemental Digital Content, http://links.lww.com/MD/N285, S3, Supplemental Digital Contents, http://links.lww.com/MD/N286, and http://links.lww.com/MD/N287). Additionally, we discovered that WDR43 expression demonstrated a robust positive association with tumor-related fibroblast infiltration in LIHC, KICH, thymoma, HNSC, HNSC-HPV+, ACC, LUAD, and GBM (Fig. 10A). Besides, WDR43 expression in LIHC was negatively correlated with macrophages (Fig. 10B).

**Figure 11.**
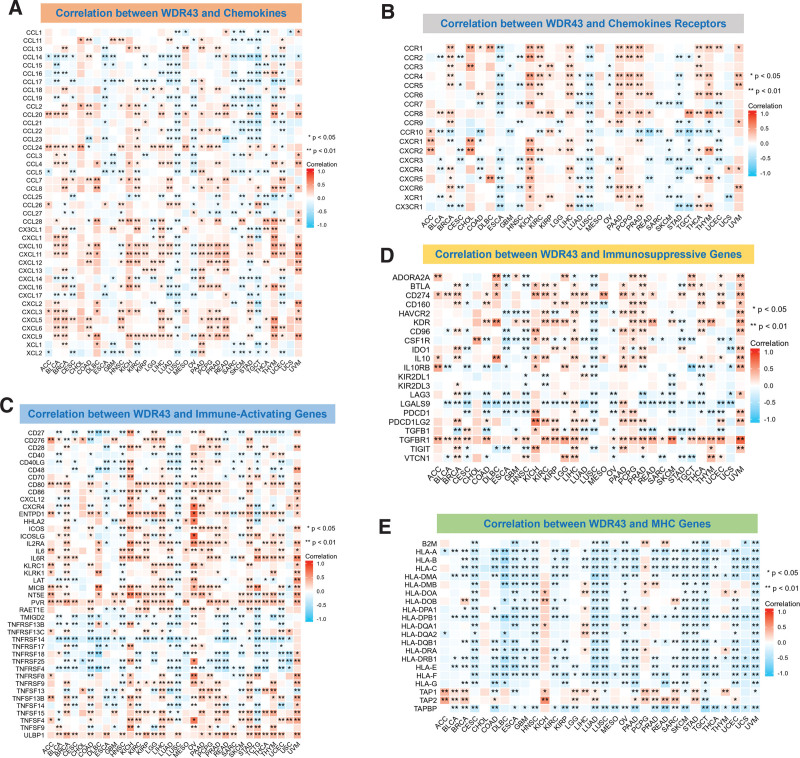
WDR43 co-expression with immune-related genes. Co-expression among WDR43 and (A) chemokines, (B) chemokine receptors, (C) immune-activated genes, (D) immunosuppressive genes, and (E) MHC genes, in which red represents a positive correlation, and blue represents a negative correlation. Genes encoding MHC, immune activation, immune suppression, chemokine, and chemokine receptor proteins were investigated. *P < .05, **P < .01, ***P < .001, and ****P < .0001. MHC = histocompatibility complex, WDR43 = WD repeat domain 43.

The study involved gene co-expression assay to investigate the association among WDR43 differential expression and immune-associated genes across 33 distinct tumor types. These genes encoded proteins such as immunosuppressive, immunological activation, MHC, chemokine, and chemokine receptor proteins. The heatmap generated from the data indicated a high degree of co-expression between WDR43 and most immune-related genes (Fig. 11). WDR43 different expression levels in various cancers were positively correlated with chemokines, including C-X-C motif chemokine ligand (CXCL) 9, CXCL10, and CXCL11. The differential expression of WDR43 demonstrated a negative association with chemokine receptors in LUSC and ESCA, whereas a positive association was detected with KICH and LIHC (Fig. 11A, B). Furthermore, most immune activation and immunosuppression genes show significant co-expression relationships in various cancers, especially in breast invasive carcinoma, LIHC, uveal melanoma, PAAD, and KICH (Fig. 11C, D). According to Figure 11E, the expression of WDR43 exhibited a negative association with almost all MHC genes in most tumors, except KICH and pheochromocytoma and paraganglioma.

### 3.7. Enrichment of WDR43-associated partners

**Figure 12.**
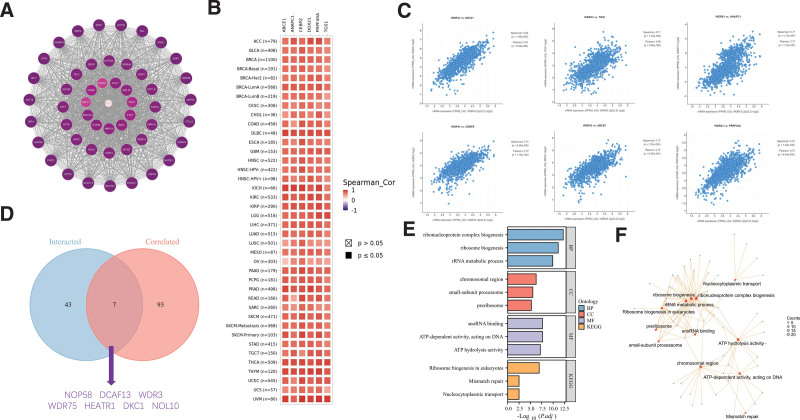
WDR43-associated gene enrichment analysis. (A) PPI network of the highest 50 WDR43 binding proteins acquired from the STRING website. (B) Heatmap correlation analysis between the expression of WDR43 and DDX21, CEBPZ, ABCE1, PRPF40A, APAPC1, and GTS1 in pan-cancer. (C) Bubble plot of correlation analysis between the expression of WDR43 and selected targeting genes in Pan-cancer. (D) A comprehensive analysis was carried out to examine the intersection between the genes correlated with WDR43-binding. (E) KEGG pathway analysis relied on the WDR43-binding and interacted genes. (F) The cnetplot for the molecular function data in GO analysis. ABCE1 = ATP binding cassette subfamily E member 1, CEBPZ = CCAAT enhancer binding protein zeta, DDX21 = DExD-box helicase 21, KEGG = Kyoto Encyclopedia of Genes and Genomes, PPI = protein–protein interactions, PRPF40A = pre-mRNA processing factor 40 homolog A, WDR43 = WD repeat domain 43.

The screening of WDR43 interacting proteins and WDR43 expression-associated genes was conducted through 2 databases, STRING and cBioPortal, and sequences of pathway enrichment analyses were conducted. Experimental data obtained through the STRING tool has revealed the existence of 50 WDR43-binding proteins. Figure 12A depicts the network of protein–protein interaction. The cBioPortal database was utilized to determine the 100 genes that exhibit the highest connection with WDR43 differential expression. According to Figures 12B, C, there was a favorable association observed among WDR43 expression levels and CCAAT enhancer binding protein zeta, anaphase-promoting complex subunit 1, ATP binding cassette subfamily E member 1, DExD-box helicase 21, trimethylguanosine synthase 1 in various tumors and pre-mRNA processing factor 40 homolog A.

Furthermore, the intersection of above 2 datasets yielded 7 members, namely DDB1 and CUL4 associated factor 13, dyskerin pseudouridine synthase 1, HEAT repeat containing 1, nucleolar protein 10, NOP58 ribonucleoprotein, WD repeat domain 3 and WD repeat domain 75 (Fig. 12D). In addition, the integration of 2 distinct datasets was performed to facilitate the GO annotation and KEGG pathway enrichment analysis. KEGG enrichment analysis outcomes revealed that the function of WDR43 in the pathogenesis of tumors may be correlated with “Ribosome biogenesis in eukaryotes,” “Mismatch repair,” and “Nucleocytoplasmic transport” (Fig. 12E). GO annotation revealed that most of these genes were associated with molecular function terms related to chromatin pathway or cell biology, including “ribosome biogenesis,” “chromosomal region,” “ATP hydrolysis activity,” and “Mismatch repair” (Fig. 12F).

**Figure 13.**
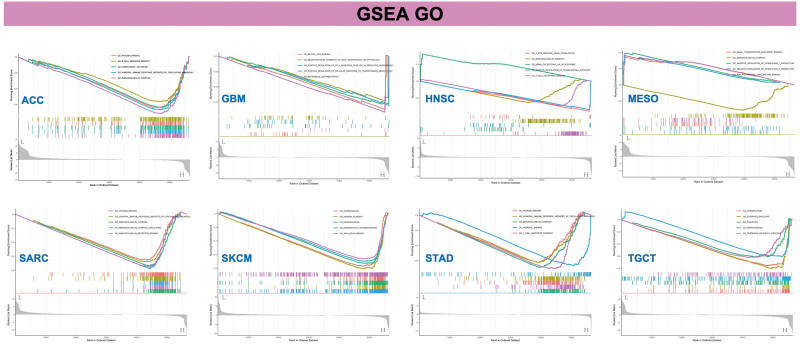
Gene set enrichment analysis of WDR43. GO functional annotations of WDR43 in ACC, GBM, HNSC, MESO, SARC, SKCM, STAD, and TGCT. Curves of distinct colors depict distinct functions or pathways that are modulated in diverse types of cancers. Peaks observed on the ascending curve demonstrate positive modulation, whereas peaks observed on the descending curve demonstrate negative modulation. ACC = adrenocortical carcinoma, GBM = glioblastoma multiforme, GO = gene ontology, HNSC = head and neck squamous cell carcinoma, MESO = mesothelioma, SARC = sarcoma, SKCM = skin cutaneous melanoma, STAD = stomach adenocarcinoma, TGCT = testicular germ cell tumor, WDR43 = WD repeat domain 43.

Furthermore, the GSEA method was employed to identify the functional enrichment patterns associated with low and high levels of WDR43 expression. As revealed in Figure 13, GO annotation revealed that WDR43 regulated immune-related functions in ACC, GBM, HNSC, MESO, SARC, skin cutaneous melanoma, STAD, and TGCT, including immune modulation, and immune response and signaling pathways, positively or negatively. Particular instances of these functions include antigen binding, immunoglobulin complex circulating, immunoglobulin complex, immunoglobulin receptor binding, B-cell receptor signal pathway, negative modulation of interleukin-1 production, T-cell receptor complex, positive modulation of translational initiation, humoral immune response promoted via circulating immunoglobulin. The findings of the KEGG pathway enrichment analysis are outlined in Figure S4, Supplemental Digital Content, http://links.lww.com/MD/N288. Our results imply that WDR43 regulates tumor immunity and inflammatory response.

## 4. Discussion

WD repeat protein exists in all eukaryotes and plays various roles in cellular processes including signal transduction, mRNA precursor processing, cytoskeleton assembly, and cell cycle regulation.^[24]^ The WD repeat protein exhibits a relationship to the occurrence of tumors, including CRC,^[25]^ NSCLC,^[26]^ estrogen receptor-negative breast cancer,^[27]^ and GBM.^[28]^ WDR43, situated on the 2p23.2 of the human chromosome, is alternatively referred to as UTP5 or NET12.^[29]^ WDR43 can function as a release factor and chromatin-associated RNA binding protein, thereby regulating pluripotency through the modulation of polymerase II activity. WDR43 can also robustly bind to promoter-associated noncoding/nascent RNA.^[11,30]^ However, several research studies have examined the molecular role of WDR43 up to this point.^[14,25]^ This study conducted an all-encompassing pan-cancer analysis of WDR43 through the entire TCGA cancers to determine its possible functions and underlying pathways through the incidence, proliferation, and clinical outcomes of distinct tumors.

The expression and prognosis of WDR43 in 33 tumors were analyzed utilizing TIMER and GEPIA databases. WDR43 was expressed heterogeneously in all TCGA cancers, with KICH and THCA showing downregulation and LUAD, GBM, HNSC, LUSC, READ, STAD, UCEC, COAD, BLCA, cholangiocarcinoma, and ESCA exhibiting overexpression. According to the IHC findings at the protein level in COAD, LIHC, UCEC, KIRC, LGG, HNSC, BRAC, and LUAD by CPTAC and HPA, WDR43 exhibited overexpression, and we confirmed it in LIHC by IHC in 48 patients of LIHC. The utilization of KM survival analysis exhibited a statistically significant correlation between increased expression levels of WDR43 and an unfavorable prognosis in PAAD, READ, esophageal squamous cell carcinoma, STAD, UCEC, LUAD, HNSC, SARC, LIHC, MESO, CESC, and KIRP. The expression level gradually increased with more advanced clinical pathological stages. A literature review revealed results similar to our studies for CRC,^[12,25]^ cervical cancer,^[31]^ breast cancer,^[32]^ and NSCLC.^[14]^ Furthermore, the nomogram revealed that WDR43 significantly impacted the prediction and demonstrated strong prognostic ability for the OS results of ACC, LIHC, and PAAD patients. The outcomes mentioned above substantiate that WDR43 has huge prospects for application as an indicator to assess various cancer prognoses.

It is widely acknowledged that somatic mutations are the root cause of all cancers.^[15]^ Most TCGA cancer types were found to have gene mutations in our study, and these mutations exhibited the highest prevalence of modification. The predominant genetic modification observed in cancers affecting WDR43 is the modifications resulting from missense mutation, with the D474N mutation situated at the Utp12 domain associated with the greatest quantity of individuals comprising the WDR43 mutation. It is widely thought that altered WDR43 is linked to worse disease-specific survival and progression-free-survival in KIRP, which raises the possibility that WDR43 genetic alterations may significantly impact various types of malignancies. However, the connection between the genetic modification of WDR43 and clinical prognosis in diverse tumors has received limited research attention.

Recently, TMB has gained significant momentum as a predictive indicator for pan-cancer through precision medicine.^[33–35]^ TMB has the potential to be used as an indicator to increase the effectiveness of immunotherapy in NSCLC^[36]^ and CRC.^[37]^ Furthermore, MSI represents a significant indicator for immune checkpoint inhibitors.^[38,39]^ According to our study, WDR43 expression is connected to MSI in 5 cancer forms, suggesting that the differential expression of WDR43 affects cancer MSI and can modulate the individual’s response to therapy involving immune checkpoint suppression. Nonetheless, a statistically significant association among the differential expression of WDR43 and TMB across pan-cancer was not detected, which may be attributed to the limitations of the dataset. The MMR system is a highly conservative mechanism of evolutionary cell repair. MSI can be triggered through modifications in the MMR system’s primary genes (MLH1, MSH2, MSH6, PMS2, EPCAM).^[40]^ There have been many reports of MMR defects being detected in Lynch syndrome-related tumors, such as CRC,^[38]^ gastrointestinal adenocarcinoma,^[41]^ and endometrial cancer.^[42]^ Most tumors exhibited an inverse association between WDR43 differential expression and MMR genes. Overall, the results of our research serve as a basis for further investigations on immunotherapy prognosis.

Indeed, it is well-established that TME characteristics impact clinical findings and are utilized as indicators for assessing the responses of the tumor cell to immunotherapy.^[43]^ Recent clinical investigations have shown that immune checkpoint inhibitor therapy significantly improved the survival prognosis for cancers with high somatic TMB levels.^[35,44]^ Tumor-infiltrating immune cells significantly affect the emergence and development of cancers in a reciprocal manner.^[45]^ The outcomes of our investigation substantiate that WDR43 expression is strongly connected to the immune cell’s biological pathways and immune-associated molecules in the most forms of cancer. The current research also showed that the genes of immunological activation, immunosuppression, MHC, chemokines, and chemokine receptor proteins were co-expressed with WDR43. The present investigation findings demonstrate that WDR43 expression is strongly linked to tumor cell immune infiltration, influences individual prognosis, and suggests potential immunosuppressive drug targets. However, further experimental investigation is required to demonstrate its function.

Additionally, GSEA revealed that increased WDR43 expression in pan-cancer may contribute to the immune-related functions, including immune regulation, immune responses, and signaling pathways. Although there is a lack of research on WDR43 in the immune system, multiple investigations have demonstrated that the above signaling pathways perform an important function in the pathogenesis or development of cancer.^[46–50]^ Overall, the abovementioned findings offer a theoretical framework for delineating the immunology and carcinogenesis of WDR43 in pan-cancer.

The present investigation exhibits certain shortcomings and restrictions that warrant recognition. First, differences in the microarray and sequencing data across different databases may introduce systematic biases. The second limitation of current investigation is the absence of experiments to validate our results, regardless of utilizing numerous bioinformatic techniques to scrutinize the correlation between WDR43 and various immunological aspects. Therefore, the diagnostic and predictive potential of WDR43 in various cancers requires further validation, especially in LIHC in a specific clinical cohort. Third, while we provided a plausible explanation for WDR43’s prognostic significance, it remains unknown how WDR43 regulates immunological activity, emphasizing the need for further research. Overall, our findings warrant further validation at both molecular and clinical levels.

## 5. Conclusions

The present investigation provided supporting evidence that WDR43 exhibited differential expression patterns across multiple forms of cancer and that its anomalous expression was connected to the advancement of tumors. In this study, we confirmed its overexpression in LIHC compared to adjacent tissues by IHC in 48 patients of LIHC. The study revealed a significant connection between WDR43 expression and various factors, including TMB, MSI, MMR, immune-associated genes, checkpoints, and immune cell infiltration. Recent findings have provided novel perspectives on implementing personalized cancer immunotherapy by revealing WDR43’s involvement in tumorigenesis and progression. Conducting prospective research that sheds light on the connection between tumor immunity and the expression of WDR43 may yield valuable findings and facilitate the advancement of immunotherapeutic approaches that target WDR43 for cancer management.

## Acknowledgments

We acknowledge our use of R software. The results are partly based upon data derived from TCGA, HPA, TIMER, GEPIA2, and Kaplan–Meier Plotter databases. We appreciate the platforms and the authors who uploaded their data.

## Author contributions

**Methodology:** Xin Yang, Ting Luo.

**Supervision:** Xin Yang, Zhuo Yang.

**Writing—original draft:** Xin Yang.

**Data curation:** Ting Luo.

**Software:** Ting Luo.

**Investigation:** Zhixin Liu.

**Resources:** Zhixin Liu.

**Writing—review & editing:** Jiao Liu.

**Conceptualization:** Zhuo Yang.

**Funding acquisition:** Zhuo Yang.

## Supplementary Material



**Figure:**
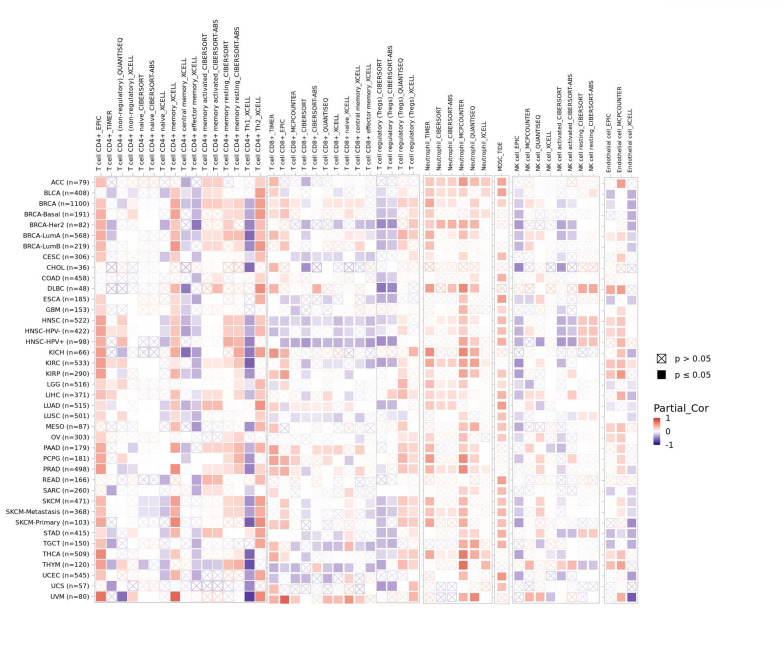


**Figure:**
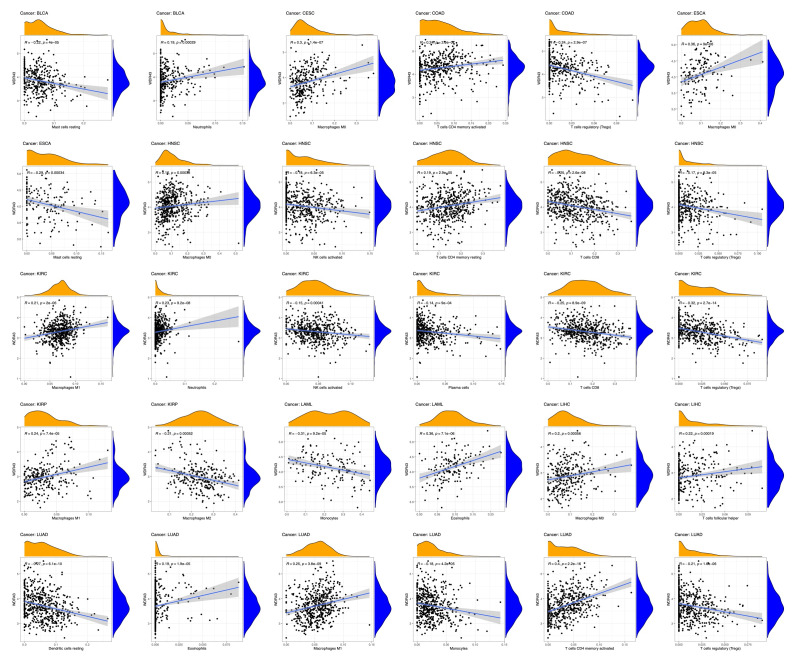


**Figure:**