# Supplementary figures and images for: WD repeat domain 43 as a new predictive indicator and its connection with tumor immune cell infiltration in pan-cancer
Source: Medicine (Baltimore). 2024 Aug 2;103(31):e39153. doi: 10.1097/MD.0000000000039153 (PMC11296459; doi:10.1097/MD.0000000000039153)

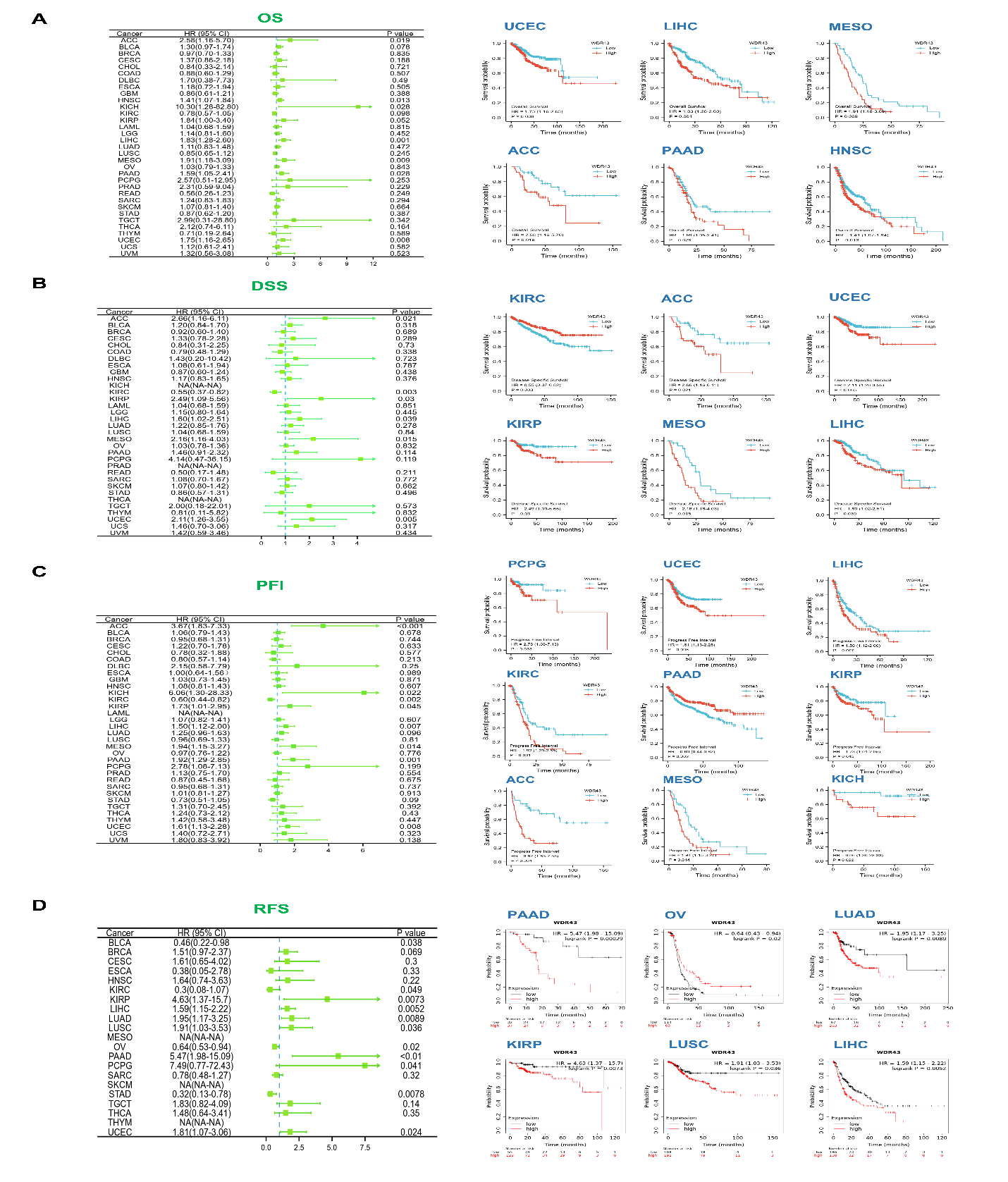

Supplement: Supplementary file 1 [file medi-103-e39153-s001.docx]

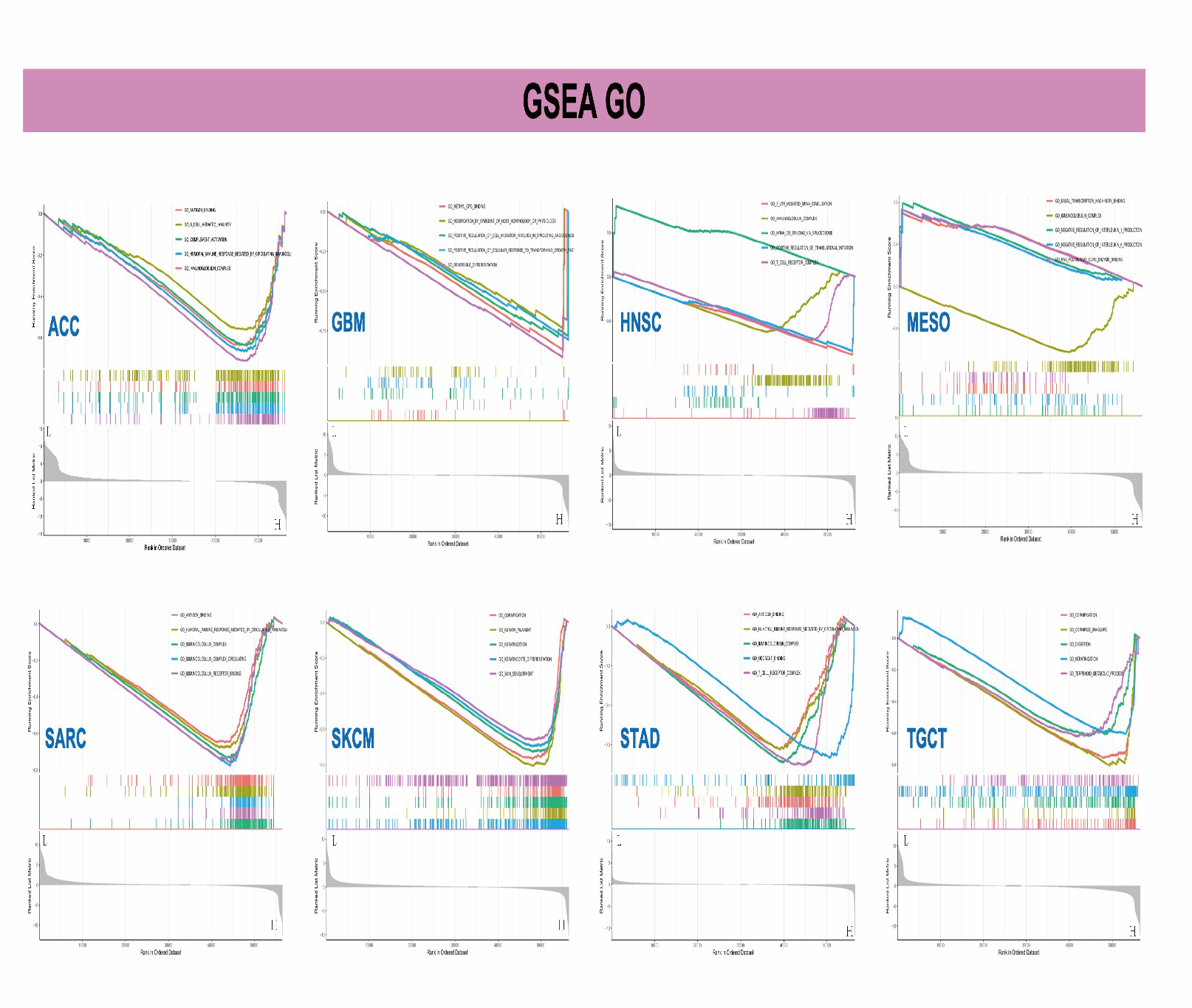

Supplement: Supplementary file 2 [file medi-103-e39153-s005.docx]
